# Unraveling the Genetic Architecture of Hepatoblastoma Risk: Birth Defects and Increased Burden of Germline Damaging Variants in Gastrointestinal/Renal Cancer Predisposition and DNA Repair Genes

**DOI:** 10.3389/fgene.2022.858396

**Published:** 2022-04-12

**Authors:** Talita Aguiar, Anne Teixeira, Marília O. Scliar, Juliana Sobral de Barros, Renan B. Lemes, Silvia Souza, Giovanna Tolezano, Fernanda Santos, Israel Tojal, Monica Cypriano, Silvia Regina Caminada de Toledo, Eugênia Valadares, Raquel Borges Pinto, Osvaldo Afonso Pinto Artigalas, Joaquim Caetano de Aguirre Neto, Estela Novak, Lilian Maria Cristofani, Sofia M. Miura Sugayama, Vicente Odone, Isabela Werneck Cunha, Cecilia Maria Lima da Costa, Carla Rosenberg, Ana Krepischi

**Affiliations:** ^1^ Department of Genetics and Evolutionary Biology, Institute of Biosciences, University of São Paulo, São Paulo, Brazil; ^2^ Human Genome and Stem Cell Research Center, Institute of Biosciences, University of São Paulo, São Paulo, Brazil; ^3^ Columbia University Irving Medical Center, New York, NY, United States; ^4^ Department of Pediatric Oncology, A. C. Camargo Cancer Center, São Paulo, Brazil; ^5^ International Center for Research, A. C. Camargo Cancer Center, São Paulo, Brazil; ^6^ GRAACC—Grupo de Apoio Ao Adolescente e Criança Com Câncer, Federal University of São Paulo, São Paulo, Brazil; ^7^ Benjamim Guimarães Foundation - Department of Pediatrics Hospital da Baleia, Belo Horizonte, Brazil; ^8^ Department of Genetics, Hospital da Criança Conceição, Hospitalar Conceição Group, Porto Alegre, Brazil; ^9^ Department Pediatric Gastroenterology, Hospital da Criança Conceição, Hospitalar Conceição Group, Porto Alegre, Brazil; ^10^ Paediatric Haemato-oncology, Hospital Santa Casa de Belo Horizonte, Belo Horizonte, Brazil; ^11^ Pediatric Cancer Institute (ITACI) at the Pediatric Department, São Paulo University Medical School, São Paulo, Brazil; ^12^ Molecular Genetics—Foundation Pro Sangue Blood Center of São Paulo, São Paulo, Brazil; ^13^ Department of Pediatric, Faculty of Medicine of the University of São Paulo, São Paulo, Brazil; ^14^ Department of Pathology, Rede D’OR-São Luiz, São Paulo, Brazil

**Keywords:** hepatoblastoma, cancer predisposition, DNA repair, MSH2, VHL, ERCC5, MUTYH, CYP1A1

## Abstract

The ultrarare hepatoblastoma (HB) is the most common pediatric liver cancer. HB risk is related to a few rare syndromes, and the molecular bases remain elusive for most cases. We investigated the burden of rare damaging germline variants in 30 Brazilian patients with HB and the presence of additional clinical signs. A high frequency of prematurity (20%) and birth defects (37%), especially craniofacial (17%, including craniosynostosis) and kidney (7%) anomalies, was observed. Putative pathogenic or likely pathogenic monoallelic germline variants mapped to 10 cancer predisposition genes (CPGs: *APC, CHEK2, DROSHA, ERCC5, FAH, MSH2, MUTYH*, *RPS19, TGFBR2* and *VHL*) were detected in 33% of the patients, only 40% of them with a family history of cancer. These findings showed a predominance of CPGs with a known link to gastrointestinal/colorectal and renal cancer risk. A remarkable feature was an enrichment of rare damaging variants affecting different classes of DNA repair genes, particularly those known as Fanconi anemia genes. Moreover, several potentially deleterious variants mapped to genes impacting liver functions were disclosed. To our knowledge, this is the largest assessment of rare germline variants in HB patients to date, contributing to elucidate the genetic architecture of HB risk.

## Introduction

The etiology of pediatric cancer is largely unknown (Saletta et al., 2015). Despite intensive research, gaps remain in our understanding of the genetic landscape of pediatric cancer susceptibility. It is assumed that germline mutations in cancer-predisposing genes (CPGs) in children and adolescents are rare events (Downing et al., 2012; Zhang et al., 2015; Kindler et al., 2018). However, the genetic predisposition to childhood cancer is most likely under-identified. Most investigations have focused on known CPGs and sequenced patients without parental samples, an approach that impairs a broad evaluation of the full range of genetic mechanisms underlying pediatric cancer risk, such as *de novo* mutations and the identification of new candidate CPGs. Recent studies of large cohorts of pediatric cancer patients have confirmed that approximately 8–18% of patients carry a germline pathogenic variant in a broad spectrum of known CPGs (Zhang et al., 2015; Ma et al., 2018a; Grobner et al., 2018; Alejandro Sweet-Cordero and Biegel, 2019; Akhavanfard et al., 2020; Capasso et al., 2020; Newman et al., 2021). These studies also highlighted that isolated factors, such as tumor type and a positive family history of cancer, have low predictive power for the presence of germline CPG mutations.

HB is the most common malignant liver tumor in the pediatric population (Heck et al., 2013), although it is considered an ultrarare disease, accounting for only 1% of all pediatric tumors (Stiller et al., 2006; Czauderna and Garnier, 2018; Feng et al., 2019). In Brazil, collected data on HB are concordant with the worldwide incidence of 0.5–1.5 cases per million (Institute Nacional de Câncer José Alencar Gomes da Silva (INCA), 2016; Aguiar et al., 2020). Most cases are diagnosed before the age of 4 years, and a male preponderance is reported (Ries et al., 1999). Nongenetic factors known to be associated with HB risk are related to very low birth weight (<1,500 g), including preterm birth (<33 weeks), small for gestational age and multiple birth pregnancies (Heck et al., 2013; Turcotte et al., 2014), and *in vitro* fertilization (Spector et al., 2019). A slow increase in HB incidence is observed in North America and Europe (Pateva et al., 2017), which can be partly due to the increased survival of children with low birth weight (Tanimura et al., 1998). An increased risk for HB development has been reported in association with a few specific genetic conditions, including Beckwith-Wiedemann syndrome (DeBaun and Tucker, 1998; Kim et al., 2017), familial adenomatous polyposis (*APC* gene) (Hirschman et al., 2005), Li-Fraumeni syndrome (*TP53* gene) (Curia et al., 2008), Aicardi syndrome (Kamien and Gabbett, 2009), and trisomy 18 (Pereira et al., 2012).

Here, we investigated the germline exome of 30 children who developed HB, 13 of whom (43%) exhibited additional clinical signs. Our analysis provides a framework for investigating candidate genes involved in HB predisposition, as well as the tumor association with specific birth defects.

## Patients and Methodology

### Participants

Thirty children diagnosed with HB were enrolled in this study, which was performed at the Institute of Biosciences, University of São Paulo, Brazil. Patients were recruited from five different Brazilian institutions, most of them (*n* = 27) from three large pediatric cancer centers of the city of São Paulo, namely, the A. C. Camargo Cancer Center, Adolescent and Child with Cancer Support Group (GRAACC), and Pediatric Cancer Institute (ITACI). In addition, three patients were recruited from other institutions, including the Hospital da Baleia (*n* = 1), Hospital da Criança Conceição (*n* = 1), and Hospital São Lucas (*n* = 1). This study was approved by the Research Ethics Committee of the Institute of Biosciences (CAAE: 09163818.4.0000.5464), and informed consent was obtained from the parents. Patient’s clinical data were documented or recovered from medical records by the oncologists who contributed to this work. Peripheral blood samples were collected from 28 patients, and normal liver tissues were recovered from two patients. Genomic samples were also obtained from 27 available parents (19 patients).

### Library Preparation and Whole-Exome Sequencing (WES)

DNA samples were extracted by phenol-chloroform followed by ethanol precipitation (Sambrook and Russell, 2006). Genomic libraries were constructed with 1 µg of genomic DNA using the following kits: Sureselect QXT V6 (Agilent Technologies), OneSeq Constitutional Research Panel (Agilent Technologies), or xGen Exome Research Panel v1.0 (IDT - Integrated DNA Technologies). The sequencing of enriched libraries was performed on the Illumina HiSeq 2,500 platform using 150 base paired end reads. The sequences were aligned to the GRCh37/hg19 human genome reference with the BWA_MEM algorithm (Li, 2013). Picard tools (v.1.8, http://broadinstitute.github.io/picard/) were used to convert the SAM file into BAM and to mark PCR duplicates. The Genome Analysis Toolkit (GATK 3.7) (McKenna et al., 2010) was used to realign indels, recalibrate the bases, and call (Unified Genotyper) and recalibrate variants (VQSR). Finally, multiallelic variants were split into different lines using the script split_multiallelic_rows.rb from Atlas2 (Challis et al., 2012) to obtain the VCF files used for analysis.

### WES Data Analysis

SNV and indel variant annotation were conducted using VarSeq software version 1.5.0 (Golden Helix) and selected by the reading depth (>10), Phred score (>20), and alternative allele frequency (>0.35). Based on the public variant databases in ABraOM (http://abraom.ib.usp.br - (Naslavsky et al., 2020), GnomAD (https://gnomad.broadinstitute.org-(Karczewski et al., 2020), and 1,000 Genomes - phase three {https://www.internationalgenome.org–(Clarke et al., 2017)}, we filtered out germline variants with frequencies below 1%, as well as those mapped to hypervariable genes (Fuentes Fajardo et al., 2012) or detected in an in-house dataset including data from 19 healthy controls.

*Coding variants* - Coding nonsynonymous missense and loss-of-function (LoF; frameshift, stop loss/gain, essential splice site, nonsense) variants were maintained for further analysis. *In silico* pathogenicity prediction for missense variants was based on six algorithms provided by the database dbNSFP (version 2.4); those predicted to be damaging to protein function by at least five different tools were prioritized. The final set of genes with rare damaging coding variants was annotated using Varelect (Stelzer et al., 2016) and HPO (Köhler et al., 2019) for ranking in association with specific phenotypes. All LoF and prioritized missense variants were validated by visual inspection of the BAM files, further annotated using the Varsome tool (Kopanos et al., 2019), and classified according to the American College of Medical Genetics and Genomics (ACMG) guidelines (David Bick et al., 2015; Hampel et al., 2015).

*Noncoding variants -* Intronic, intergenic, 3′ and 5’ untranslated regions (UTRs), and splice region variants were annotated using SNPnexus v4 (Oscanoa et al., 2020), a web-based annotation tool for the analysis and interpretation of variants that includes databases of regulatory elements and regions, such as miRbase (ftp://mirbase.org/pub/mirbase/20/genomes/), Vista HMR (http://hgdownload.cse.ucsc.edu/goldenPath/hg19/database/), and ENCODE (ftp://ftp.ensembl.org/pub/grch37/release-95/mysql/regulation_mart_95/); phenotype and disease association (Genetic Association of Complex Diseases and Disorders (GAD) - http://hgdownload.cse.ucsc.edu/goldenPath/hg19/database/, ClinVar, and COSMIC); and noncoding scoring (Combined Annotation Dependent Depletion (CADD) - http://cadd.gs.washington.edu/ download, Fitness Consequences of Functional Annotation (fitCons)-http://compgen.cshl.edu/fitCons/0downloads/tracks/V1.01/i6/scores/; and Chromatin Effects of Sequence Alterations (DeepSEA)-http://deepsea.princeton.edu/help/).

*Copy number variants (CNVs) -* CNVs and region of homozygosity (ROH) events were derived from WES data using the software Nexus Copy Number 9 (Biodiscovery) with the SNP-FASST2 segmentation algorithm (threshold log_2_ Cy3/Cy5 ratio of |0.3| for gains and losses; minimum ROH length of 5 Mb). Common CNVs (Database of Genomic Variants, http://dgv.tcag.ca/dgv/app/home) were disregarded. For CNV validation, chromosome microarray analysis (CMA) was performed using the 180K platform (Agilent Technologies), as previously reported (Oliveira et al., 2018).

## Results

### Clinical Characterization of the HB Cohort

The clinical features of the 30 patients who developed HB are described in Table 1; the details of their tumors can be found in Supplementary Table S1. The mean age at HB diagnosis was 24 months, excluding one patient who was diagnosed at 17 years (P07). Twenty-one patients were male (70%), which agrees with the literature on sex bias in HB (Spector and Birch, 2012; Feng et al., 2019). Six patients (∼20%) presented with a family history of cancer (relatives of different degrees developed different tumors at varying ages).

**TABLE 1:** Clinical description of the 30 cases of patients who developed hepatoblastoma.

ID	Gender	Diagnosis Age	Premature	Deceased	Associated Clinical Conditions	Familial Clinical History
P01	M	42 months	N/A	...	...	...
P02	M	12 months	...	...	Chronic hypomagnesemia and growth hormony deficiency	...
P03	F	18 months	N/A	Yes	...	...
P04	M	9 months	...	...	...	cancer (paternal uncle - leukemia)
P05	F	36 months	...	...	...	...
P06	M	9 months	...	...	...	...
P07	M	17 years	...	Yes	Congenital hepatomegaly	...
P08	M	54 months	...	Yes	...	...
P09	M	30 months	...	...	Renal dysfunction (non-functional left kidney)	...
P10	F	36 months	...	...	Idiopathic early telarca	...
P11	F	1 month	...	...	Syndromic - congenital HB and unilateral renal agenesis	cancer (maternal grandmother - breast; paternal great-grandfather - prostate)
P12	M	19 months	…	…	…	cancer (paternal great-grandfather melanoma and maternal great-grandfather lung (smoker)
P13	M	28 months	Yes	...	Syndromic—craniosynostosis, facial dysmorphisms, developmental delay	deceased older sisther (prematurity)
P14	M	7 months	...	...	...	...
P15	M	7 months	...	...	...	...
P16	M	12 months	...	...	...	...
P17	F	58 months	...	Yes	Syndromic—Hirschsprung disease, protruding ears, hypertelorism, nail dysplasia, developmental delay	
P18	M	17 months	Yes	...	Syndromic—Hirschsprung disease, congenital ileal atresia, congenital bilateral cataract, sensorineural deafness, developmental delay	older sister—congenital biletaral cataract; older brother - intestinal atresia (terminal ileum)
P19	M	2 months	...	...	...	...
P20	M	7 months	Yes	...	Metabolic bone disease	...
P21	M	5 months	...	...	Congenital HB	...
P22	M	6 months	Yes	...	...	...
P23	M	9 months	...	...	...	...
P24	F	15 months	Yes	...	Syndromic—craniofacial dysmorphisms, nail dysplasia, developmental delay	...
P25	M	20 months	...	...	...	...
P26	F	8 months	...	...	...	...
P27	F	6 years	...	...	...	cancer (familial adenomatous polyposis - mother, sister, maternal uncle)
P28	M	4 months	...	...	Syndromic—craniosynostosis and developmental delay	...
P29	M	17 months	…	...	Syndromic—postnatal microcephaly, congenital malformations of the VACTERL spectrum, developmental delay	cancer (paternal cousin - central nervous system in childhood); maternal schizophrenia
P30	F	15 months	Yes	...	Syndromic—craniofacial dysmorphisms, short neck, polysyndactyly, laryngomalacia, severe bronchodysplasia, swallowing disorder, developmental delay	cancer (several family members); paternal cousin with polysyndactyly

F: female; M: male.

N/A- Not available; CPG, cancer predisposition gene; AD, autosomal dominant Inheritance; AR, autosomal recessive inheritance.

Sixteen patients were diagnosed with high-risk HB, classified according to the CHIC stratification (Czauderna et al., 2016; Meyers et al., 2017), and 10 presented pulmonary metastasis at diagnosis. Except for P23, who underwent surgery at diagnosis, all patients received neoadjuvant chemotherapy protocols followed by tumor resection or transplantation. Eight patients received a liver transplant, and two relapsed. Four patients died from the disease.

Six out of the 30 patients were born prematurely (<37 weeks), corresponding to 20% of the group. Eleven patients had birth defects (37%), and eight of them were classified as syndromic due to the presentation of two or more congenital clinical features. Among them, five patients had craniofacial anomalies, two of whom were diagnosed with craniosynostosis (P13 and P28); two patients were born with kidney anomalies (P11 and P09); and P07, who was diagnosed with HB at an advanced age (17 years), was born with mild hepatomegaly.

P11, female, had a congenital HB diagnosed at 1 month of age; in addition, this patient was born with unilateral renal agenesis. P13, a male, was born extremely premature at 27 weeks. More details about the clinical features of both patients can be found in our previous study (Aguiar et al., 2020).

Two patients were born with Hirschsprung disease and other clinical features (P17 and P18). P17, female, was the third child of no consanguineous parents. She was born at term, and her two siblings had a normal phenotype. Abdominal volume and no evacuation were detected in the first 24 h after birth, and the diagnosis of Hirschsprung disease was made. In the clinical evaluation at 5 years old, she presented with global neuropsychomotor delay, facial dysmorphisms, clinodactyly, and nail dysplasia (hypoplastic). HB was diagnosed at the age of four and classified as an epithelial subtype with a predominance of embryonal cells, PRETEXT IV, high risk. She underwent the SIOPEL six chemotherapy protocol and died before the surgical procedure. P18, male, was the third child of a consanguineous couple; his sister was born with congenital bilateral cataracts, while his brother exhibited intestinal atresia-terminal ileus. The patient was born prematurely (28 weeks), with a syndromic phenotype composed of congenital ileal atresia, bilateral cataracts, and sensorineural deafness. His mother, who had gestational risk (cardiac defect and preeclampsia), died during his birth due to congestive heart failure. HB tumors were diagnosed at 1 year of age and classified as fetal epithelial subtype, PRETEXT II, and low risk. He underwent a chemotherapy protocol with cisplatin, doxorubicin, and ifosfamide, followed by partial hepatectomy. Currently, the patient is in post treatment follow-up, and clinical details have been previously published (Pinto et al., 2016).

P24 had facial dysmorphisms and dysplastic nails of the hands and feet, in addition to developmental delay. P29 was born with congenital malformations of the VACTERL spectrum and exhibited postnatal microcephaly and developmental delay. P30 presented craniofacial dysmorphisms, turricephaly, short neck, laryngomalacia, swallowing disorder, severe bronchodysplasia, and polysyndactyly of the right fifth digit, in addition to severe malnutrition and neuropsychomotor delay.

We performed comparisons between disease risk status and other variables (gender, premature birth, death, associated clinical conditions, familial clinical history, and age at diagnosis). When only categorical variables were considered, the non-parametric chi-square test with Yates continuity correction and Fisher exact test were applied to determine if the distribution of the data differed statistically from random expectations (null hypothesis). In cases in which continuous variables were considered, we performed the non-parametric Kruskal–Wallis test. Although no significant results were found, mainly due to the small size of the sample, when we compared the distribution of individuals with craniofacial or kidney defects according to risk stratification, a marginally significant result was obtained (*p*-value = 0.10), which can indicate a tendency of individuals with high-risk status to be more likely to present craniofacial defects, while individuals with less severe status are more prone to have kidney defects (Supplementary Figure S1).

### Germline Coding and Noncoding Variants (SNVs and Indels)

**FIGURE 1:**
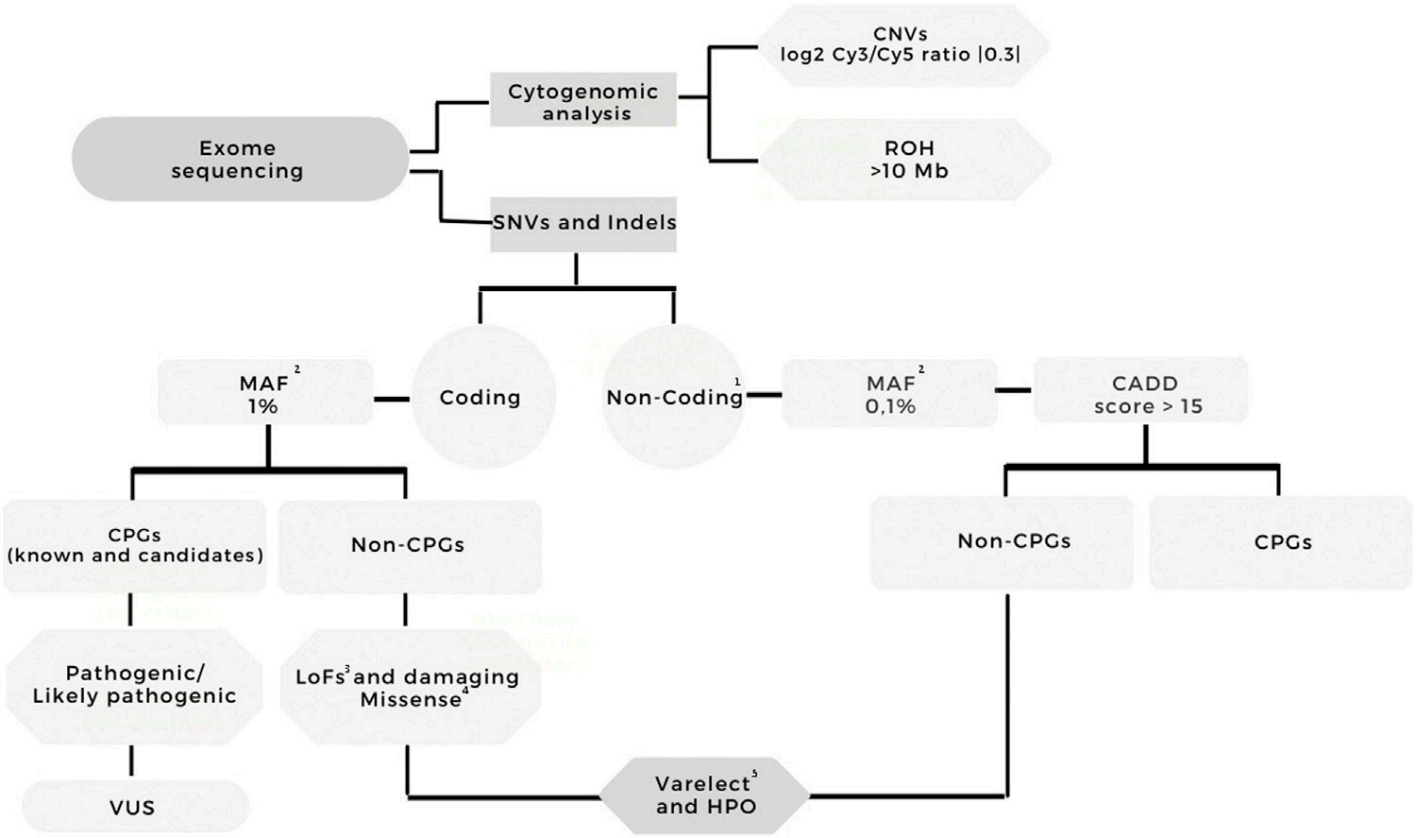
Workflow of the exome sequencing analysis. Variants were filtered according to quality (Phred score >20, read depth >10, variant allele frequency >35%); coding and noncoding variants were separately analyzed; frequency (GnomAD, ABraOM, 1K Genomes); the effect on coding variants (frameshift, stop loss/gain, missense, splice site, nonsense); for missense variants, the prediction of pathogenicity in at least five out of six algorithms; HPO annotation (hepatoblastoma, abnormalities of the liver, and cancer). The filtered variants were visually examined using Integrative Genomics Viewer (IGV) software (http//www.broadinstitute.org/igv) to further filter out possible strand bias and homopolymeric region artifacts. All the filtered variants mapped to cancer predisposition genes were classified using the ACMG guidelines. One- Intronic variants, 3′UTR, 5′UTR; two- MAF: GnomAD, ABraOM, 1K Genomes; three- Frameshift, stop loss, stop gain, missense, splice site, nonsense variants; four- Missense variants with dbNSFP Functional Prediction of pathogenicity in at least five out of six algorithms; five- Terms used for Varelect and HPO annotation: Hepatoblastoma, abnormalities of the liver, and cancer. CNV—copy number variation, ROH - region of homozygosity, MAF—maximum allele frequency, CPG—cancer predisposition gene, VUS—variant of uncertain significance, HPO—Human Phenotype Ontology, HB—hepatoblastoma.

Figure 1 summarizes the analysis workflow of the WES data. The mean sequencing depth of the exomes was 92× (Supplementary Table S2 presents sequencing metrics and the type of genomic library for each sample); P03 was the only sample presenting 10x on-target coverage below 80%.

**FIGURE 2:**
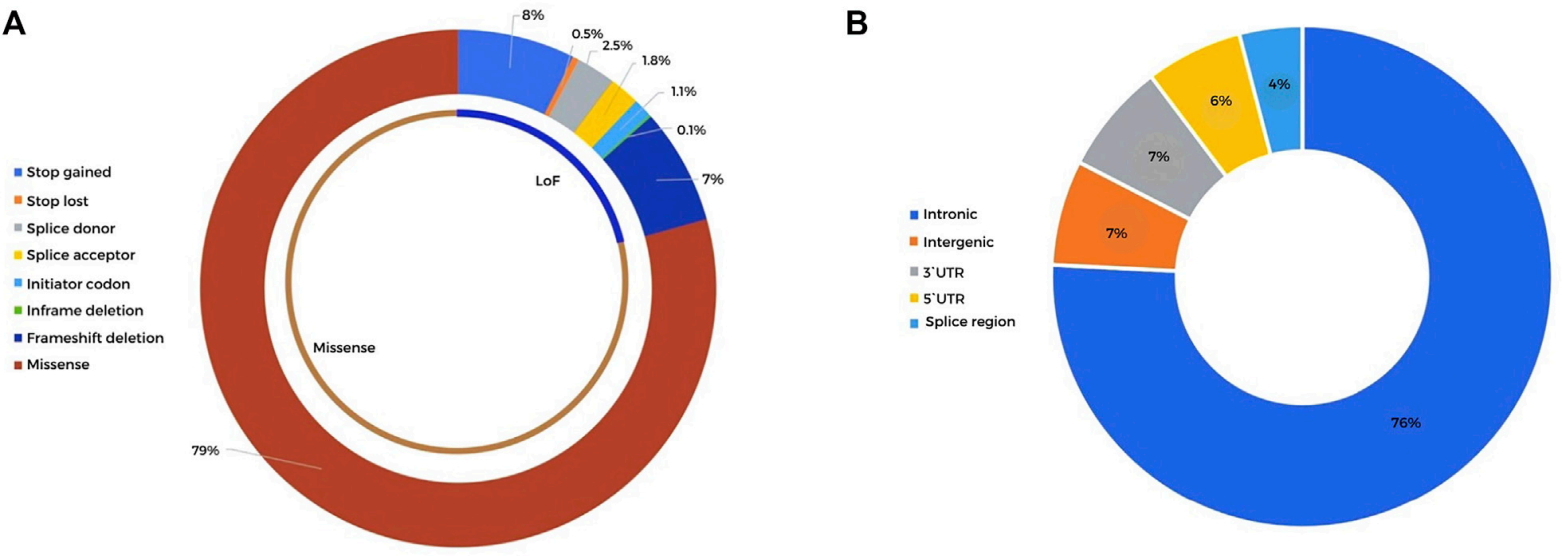
Distribution of the detected high-quality rare coding and noncoding variants detected in 30 HB patients **(A)**. Sequence ontology of the rare coding variants detected after selection by the read depth (>10), Phred score (>20), alternative allele frequency (>0.35), and population frequency (<1%). A total of 2,107 variants were classified into 1,671 missense mutations and 436 LoF variants **(B)**. Sequence ontology of the rare noncoding variants detected after selection by the read depth (>10), Phred score (>20), alternative allele frequency (>0.35), and population frequency (<0.1%). A total of 2,070 noncoding variants were distributed in intronic, intergenic, and 3′ and 5′ UTRs.

A total of 9,467 rare (population frequency <1%) germline coding nonsynonymous variants were detected in the cohort of 30 HB patients, mapping to 6,102 genes. Details of all variants can be found in Supplementary Table S3. A total of 2,107 of these rare variants, related to 1,737 different genes and including 1,671 missense mutations and 436 LoF variants (Figure 2A), met our criteria of a read depth >10, Phred score >20, and alternative allele frequency >0.35. Pathogenic (P) or likely pathogenic (LP) variants mapped to morbid OMIM genes that could explain the syndromic phenotypes of some patients were not detected.

Using a list of 222 CPGs composed of 119 known CPGs (49 of them reported in OMIM), in addition to 103 candidates that were compiled by revision of recent publications (Supplementary Table S4), we investigated the presence of rare coding variants that could be related to cancer development. No homozygous or compound heterozygous pathogenic (P) or likely pathogenic (LP) variants were observed in known or candidate CPGs. Eleven heterozygous putative P/LP variants mapped to ten CPGs were detected in ten patients, comprising 33% of the group (Table 2; details provided in Supplementary Table S5); *VHL* variants were detected in two patients. Among the 10 patients with potentially P/LP variants in CPGs, only four presented a family history of cancer; four of them were syndromic, and three were born prematurely. One of these patients (P28) carried two variants mapped to known recessive CPGs. Eight of these variants were detected in seven autosomal-dominant CPGs (known or candidate), including an *APC* LoF variant (OMIM #175100 FAMILIAL ADENOMATOUS POLYPOSIS one; gastrointestinal carcinomas) and six missense variants, mapped to the *CHEK2* (LI-FRAUMENI SYNDROME two; colorectal, breast and prostate cancer)*, DROSHA* (50)*, MSH2* (LYNCH SYNDROME I; colorectal cancer/MISMATCH REPAIR CANCER SYNDROME two; hematologic malignancy, brain tumors, and gastrointestinal tumors)*, RPS19* (DIAMOND-BLACKFAN ANEMIA one; osteogenic sarcoma, myelodysplastic syndrome, colon cancer), *VHL* (VON HIPPEL-LINDAU SYNDROME; renal cell carcinoma, pheochromocytoma, hemangioblastoma, hypernephroma, pancreatic cancer, paraganglioma, adenocarcinoma of the ampulla of Vater)*,* and *TGFBR2* (COLORECTAL CANCER, HEREDITARY NONPOLYPOSIS, TYPE 6) genes. Three variants were detected in three CPGs associated with recessive conditions: an *ERCC5* LoF (XERODERMA PIGMENTOSUM, COMPLEMENTATION GROUP G; skin cancers) and missense variants mapped to *FAH* (TYROSINEMIA, TYPE I; hepatocellular carcinoma) and *MUTYH* (FAMILIAL ADENOMATOUS POLYPOSIS two; colorectal carcinomas).

**TABLE 2:** Description of 11 potentially pathogenic/likely pathogenic germline heterozygous variants mapped to 10 known/candidate cancer predisposition genes and detected in 10 hepatoblastoma patients.

ID	Gene	OMIM Condition	CPG category	Genomic Coordinates (GRCh37)	HGSV Nomenclature	Frequency (GnomAD exomes/AbraOM)	Protein Change	Pathogenicity Assessment	Inheritance	Clinical Information	Literature Related to Cancer Predisposition Evidence
P03	*TGFBR2*	#614331 COLORECTAL CANCER, HEREDITARY NONPOLYPOSIS, TYPE 6/#610168 LOEYS-DIETZ SYNDROME 2 (AD)	bona fide	3:30733054	NM_001024847.2:c.1742A > G	...	p.Lys581Arg	VUS/likely pathogenic	maternal	...	Xu and Pasche, (2007) Ma et al. (2012) Felicio et al. (2021)
P04	*DROSHA*	...	candidate	5:31424577	NM_013235.5:c.3218A > C	0.000016/0	p.Asp1073Ala	VUS/likely pathogenic	N/A	Positive familial cancer history	Wegert et al. (2015) Carraro et al. (2016) Felicio et al. (2021)
P11	*FAH*	#276700 TYROSINEMIA, TYPE I (AR)	bona fide	15:80460444	NM_000137.2:c.506C > T	...	p.Ser169Phe	Likely pathogenic	maternal	Positive familial cancer history; syndromic	Zhang et al. (2015) Carta et al. (2020) Capellini et al. (2021)
P13	*MSH2*	#120435 LYNCH SYNDROME I/#158320 MUIR-TORRE SYNDROME (AD); #619096 MISMATCH REPAIR CANCER SYNDROME 2 (AR)	bona fide	2:47630464	NM_000251.2:c.134C > A	...	p.Ala45GLu	VUS/likely pathogenic	maternal	Premature; syndromic	Capasso et al. (2020)
P16	*VHL*	#193300 VON HIPPEL-LINDAU SYNDROME/#171300 PHEOCHROMOCYTOMA (AD)	bona fide	3:10183772	NM_000551.3:c.241C > T	0.000218/0	p.Pro81Ser	Likely pathogenic (modifier)	N/A	...	Schmidt et al. (2016)
P21	*CHEK2*	#609265 LI-FRAUMENI SYNDROME 2/#14480 BREAST, #114500 COLORECTAL, # 176,807 PROSTATE SUSCEPTIBILITY TO CANCER	bona fide	22:2,9091741	NM_007194.4:c.1216C > T	0.000060/0	p.Arg406Cys	VUS/likely pathogenic	not maternal	Congenital tumor	Vahteristo et al. (2002) Bąk et al. (2014) Southey et al. (2016)
P22	*VHL*	#193300 VON HIPPEL-LINDAU SYNDROME/#171300 PHEOCHROMOCYTOMA (AD)	bona fide	3:10183685	NM_000551.3: c.154G > A	0.000085/0	p.Glu52Lys	VUS/likely pathogenic	maternal	Premature	Schmidt et al. (2016)
P27	*APC*	#175100 FAMILIAL ADENOMATOUS POLYPOSIS 1 (AD)/#135290 DESMOID disease, HEREDITARY (AD)	bona fide	5:112175038	NM_000038.6:c.3747C > A	...	p.Cys1249*	Pathogenic	maternal	Positive familial cancer history*	Baeg et al. (1995) Schmidt et al. (2016)
P28	*ERCC5*	# 616,570 CEREBROOCULOFACIOSKELETAL SYNDROME 3 (AR)/# 278,780 XERODERMA PIGMENTOSUM, COMPLEMENTATION GROUP G (AR)	bona fide	13:103514580	NM_000123.3:c.1081delC	...	p.Leu361Trpfs*18	Likely pathogenic	not paternal	Syndromic	Fassihi et al. (2016)
	*MUTYH*	#608456 FAMILIAL ADENOMATOUS POLYPOSIS 2 (AR)	bona fide	1:45798111	NM_001048174.1:c.656G > A	0.000008/0	p.Arg219GLn	VUS/likely pathogenic	paternal		Barreiro et al. (2022)
P30	*RPS19*	# 105,650 DIAMOND-BLACKFAN ANEMIA 1 (AD)	bona fide	19:42365273	NM_001022.3:c.164C > T	0.000248/0	p.Thr55Met	VUS/likely pathogenic	maternal	Positive familial cancer history; premature; syndromic	Luft, (2010)

**FIGURE 3:**
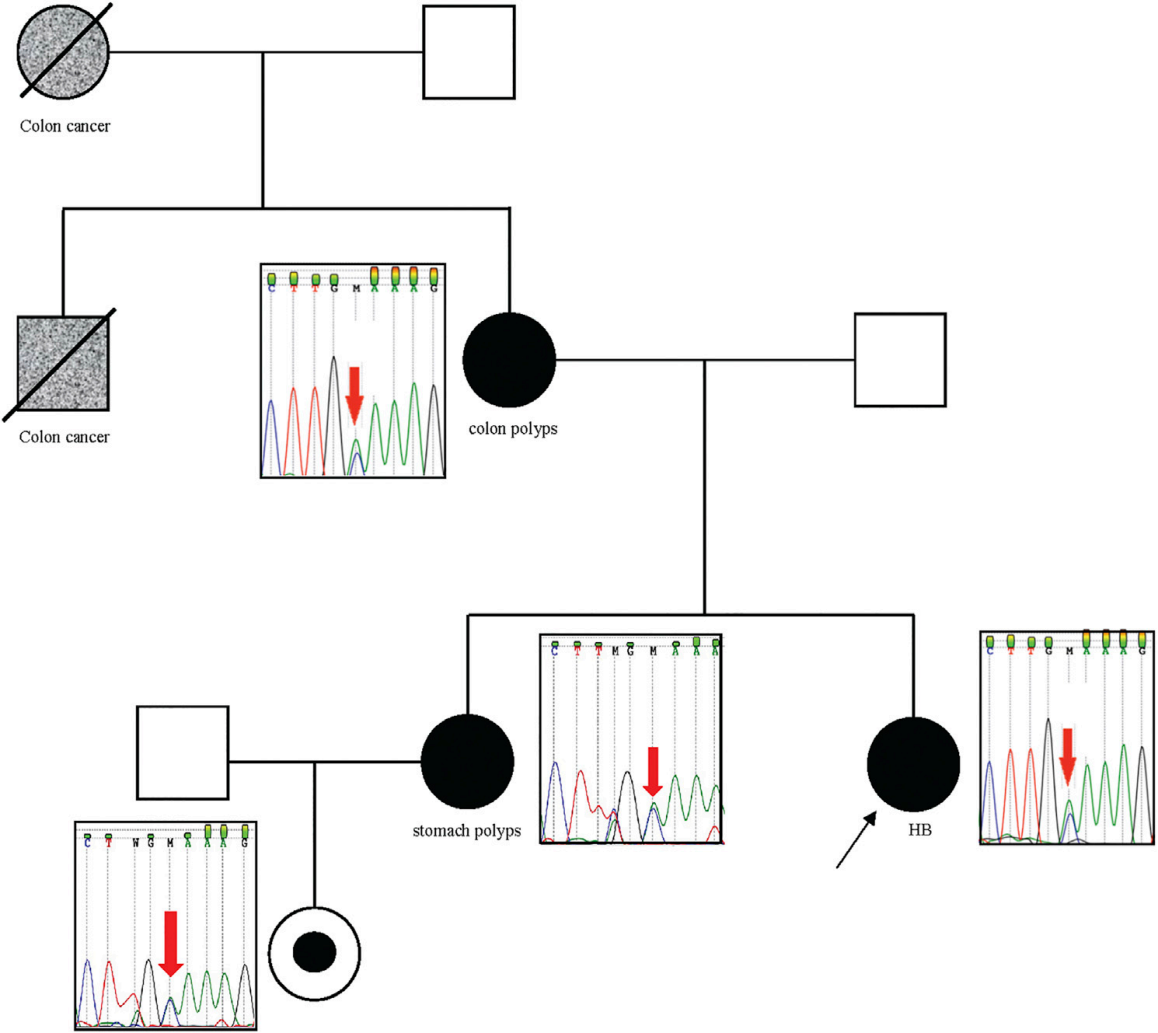
*APC* pathogenic variant segregates with the cancer phenotype in the family of patient P27. The patient P27 is indicated with the black arrow. P27, her mother, sister, and niece are carriers of the pathogenic p. Cys1249* variant in the *APC* gene, which was identified by exome sequencing in the patient, and validated by Sanger sequencing in their indicated relatives. Her mother and sister had colon polyps and stomach polyps, respectively. The maternal grandmother and uncle had colon cancer (both are dead).

We investigated genomic data available of tumor tissues derived from three of these patients with relevant germline variants described in Table 2 (P03, P13, P21); however, additional somatic mutations in the same gene were not observed in these cases, and tumor tissue from patient P13 was no longer available to test microsatellite instability. In particular, Patient P27, who carries an *APC* pathogenic variant, had a strong familial cancer history, in which her mother and sister were diagnosed with familial adenomatous polyposis (FAP), and her maternal grandmother and uncle, already dead, had colon cancer. Sanger sequencing analysis confirmed the segregation of the detected pathogenic *APC* variant with the cancer phenotype in this family (Figure 3).

In addition, 44 VUS mapped to 34 known/candidate CPGs were observed in 21 patients (70%) (Table 3), most of whom carried more than one variant. VUS mapped to the *ATM*, *BRCA2*, *COL7A1*, *DHCR7*, *DOCK8*, *FANC*s, and *GLI3* genes were detected in more than one patient.

**TABLE 3:** Description of 44 germline rare heterozygous variants of uncertain significance (VUS) mapped to known/candidate cancer predisposition genes detected in 21 hepatoblastoma patients.

ID	Gene	Inheritance Pattern	CPG category	Genomic Coordinates*	HGSV Nomenclature	Variant Frequency (GnomAD)	Protein Change	ClinVar (Classification/ID)	Variant Inheritance
P09	*ATM*	Recessive; Dominant	bona fide	11:108121787	c.1595G > A	0.0002	p.Cys532Tyr	LB (3); VUS(7)/133603	n/a
P29	*ATM*	Recessive; Dominant	bona fide	11:108198394	c.6998C > A	0.00009	p.Thr2333Lys	LB (4); VUS(2)/127434	paternal
P15	*BARD1*	Dominant	bona fide	2:215645351	c.1247T > G	...	p.Leu416Arg	VUS/237815	not maternal
P11	*BRCA2*	Recessive; Dominant	bona fide	13:32912346	c.3858_3860delAAA	0.00009	p.Lys1286del	B (1); LB (9); VUS(1)/37861	not maternal
P12	*BRCA2*	Recessive; Dominant	bona fide	13:32906711	c.1096T > G	0.00003	p.Leu366Val	LB (3); VUS(4)/89040	Paternal
P02	*COL7A1*	Recessive; Dominant	bona fide	3:48629243	c.1370C > T	0.00003	p.Pro457Leu	VUS/345877	maternal
P28	*COL7A1*	Recessive; Dominant	bona fide	3:48628112	c.1774C > T	0.00004	p.Arg592Cys	...	maternal
P02	*DHCR7*	Recessive	candidate	11:71152316	c.583G > A	0.00002	p.Ala195Thr	...	maternal
P09	*DHCR7*	Recessive	candidate	11:71146861	c.988G > A	0.0004	p.Val330Met	VUS/420113	n/a
P26	*DHCR7*	Recessive	candidate	11:71146861	c.988G > A	0.0004	p.Val330Met	VUS/420113	maternal
P30	*DIS3L2*	Recessive	bona fide	2:233201216	c.2534A > G	...	p.Gln845Arg	...	maternal
P05	*DOCK8*	Recessive	bona fide	9:432,299	c.4760T > C	0.00005	p.Met1587Thr	VUS/835366	maternal
P16	*DOCK8*	Recessive	bona fide	9:328,086	c.959C > T	0.000004	p.Thr320Met	VUS/942058	maternal
P30	*DOCK8*	Recessive	bona fide	9:336,712	c.1416C > G	0.00002	p.Phe472Leu	...	maternal
P13	*EP300*	Dominant	candidate	22:41565569	c.4235C > T	0.00002	p.Ala1412Val	...	paternal
P17	*ERCC2*	Recessive	bona fide	19:45868145	c.545C > T	0.00062	p.Ala182Val	B (1); LB (1); VUS(1)/	maternal
P26	*EXT2*	Dominant	bona fide	11:44165865	c1242 G > A	0.000062	p.Trp414*		not maternal
P24	*FANCA*	Recessive	bona fide	16:89811469	c.3524C > T	0.00032	p.Pro1175Leu		maternal
P10	*FANCD2*	Recessive	bona fide	3:1,0132002	c.3710T > C	0.000008	p.Val1237Ala	VUS/578150	not maternal
P29	*FANCD2*	Recessive	bona fide	3:10107551	c.2273G > C	0.00058	p.Cys758Ser	LB (1); VUS(2)/456351	maternal
P05	*FANCG*	Recessive	bona fide	9:35076975	c.770G > A	0.000004	p.Arg257His	VUS/802484	not maternal
P09	*FANCM*	Dominant	candidate	14:45654489	c.4585G > A	...	p.Asp1529Asn	...	n/a
P23	*FANCM*	Dominant	candidate	14:45644816	c.2859A > C	...	p.Lys953Asn	LB (1); VUS(6)/313209	maternal
P29	*FAS*	Dominant	bona fide	10:90773115	c.667A > C	0.00009	p.Asn223His	VUS/570775	maternal
P08	*FGFR3*	Dominant	candidate	4:1801029	c.158G > C	0.00002	p.Ser53Thr	VUS/501373	n/a
P16	*FH*	Recessive; Dominant	bona fide	1:241671977	c.664T > A	0.00003	p.Ser222Thr	VUS/576908	paternal
P21	*FLCN*	Dominant	bona fide	17:17129602	c.284A > G	...	p.Tyr95Cys	...	maternal
P24	*GBA*	Recessive	bona fide	1:155206060	c.1200G > A	0.0003	p.Met400Ile	...	maternal
P18	*GLI3*	Dominant	candidate	7:42187947	c.245G > A	0.00001	p.Arg82Lys	...	paternal
P28	*GLI3*	Dominant	candidate	7:42187959	c.233C > T	0.000008	p.Ser78Leu	...	maternal
P04	*JMJD1C*	Dominant	candidate	10:64949103	c.6395A > T	0.001	p.Lys2132Ile	LB/571988	maternal
P23	*KDR*	Dominant	candidate	4:55963918	c.2525G > A	...	p.Arg842His	...	maternal
P30	*MET*	Dominant	bona fide	7:116395422	c.1715G > A	0.000501,263	p.Ser572Asn	B (3); LB (3)​/188358	maternal
P29	*MRE11*	Recessive	candidate	11:94180501	c.1667A > G	0.0001	p.Asn556Ser	LB (1); VUS(5)/142425	maternal
P29	*MSH6*	Recessive; Dominant	bona fide	2:48018139	c.334A > G	...	p.Asn112Asp	VUS/220150	paternal
P04	*NDRG4*	Unknown	candidate	16:58538132	c.358G > A	0.00003	p.Val120Met	...	not maternal
P22	*NYNRIN*	Recessive	candidate	14:24886448	c.5493G > C	...	p.Gln1831His	...	maternal
P22	*PTCH1*	Dominant	bona fide	9:98231286	c.1997C > T	0.00001	p.Thr666Met	VUS/820499	maternal
P23	*RECQL*	Dominant	candidate	12:21636438	c.572C > A	...	p.Pro191GLn	...	paternal
P29	*RECQL4*	Recessive	bona fide	8:145741407	c.1096G > C	0.000004	p.Gly366Arg	VUS/239691	paternal
P30	*RHBDF2*	Dominant	bona fide	17:74475818	c.356G > A	0.000004	p.Ser119Asn	...	paternal
P23	*SH2B3*	Recessive	candidate	12:111856571	c.622G > C	...	p.Glu208GLn	VUS/30445	paternal
P22	*SLX4*	Recessive	bona fide	16:3634797	c.4712C > T	0.00002	p.Thr1571Met	...	maternal
P12	*TERT*	Recessive; Dominant	bona fide	5:1293676	c.1323_1325delGGA	0.002	p.Glu441del	B (1); LB (9); VUS(3)/212398	paternal

*Genomic coordinates given according to GRCh37.

N/A- Not available; CPG, cancer predisposition gene; B- Benign; LB, Likely benign; VUS, Variant of uncertain significance.

Thirty-two genes related to liver differentiation or function were found to be affected by rare variants (*ABCB11, ABCB4, ABCC2, ABCC3, AFP, ALB, CYP1A1, CYP1A2, CYP2C19, CYP2C8, CYP2C9, CYP2D6, CYP3A7, FAH, FOXA2, KRT7, KRT8, MET, NR1I2, ONECUT1, PAH, POU5F1, PPARG, SOX17*) (Supplementary Table S6).

We also investigated whether the observed sex bias in the group could be explained by an increased burden of rare damaging variants in one of the sexes; we did not detect significant differences considering the average of rare damaging variants in male and female patients (∼66.7 and 68.1, respectively), LoF variants (∼15 variants in both groups), and rare damaging CPG variants (∼8 and 6, respectively).

A total of 2,069 noncoding variants passed our filters (read depth >10, Phred score >20, alternative allele frequency >0.35, frequency in population databases >0.1%), including intronic (76%), intergenic (7%), 3′ prime UTR (7%), 5′ prime UTR (6%), and splice region (4%) variants (Supplementary Table S7; Figure 2B). These variants were annotated using SNP Nexus (Oscanoa et al., 2020), and those with CADD scores above 15 and associated with cancer (https://geneticassociationdb.nih.gov/) were prioritized for further analyses. Table 4 details the 23 prioritized rare noncoding variants. Two variants were observed in the intronic regions of the CPGs *BRAF* and *CREBBP*, but with no evidence of a functional effect. In particular, P12 carried a *de novo* mutation in the 5’ UTR of the *TCF7* gene, an important effector protein in the Wnt pathway; this patient also carries a paternally inherited coding *TCF7* VUS (c.1060C > G).

**TABLE 4:** Rare germline non-coding variants detected in HB patients which were prioritized (CADD score above 15 and association with cancer).

ID	Gene	Genomic Coordinates*	Ref/Alt	Sequence Ontology	Variants	CADD Score	Inheritance	Co-Occurrence with Coding Variant
**P02**	*KLF12*	13:74518363	T/A	intron_variant	c.34–156A > T	15.12	N/A	
**P02**	*PRKCH*	14:61788795	C/T	5_prime_UTR_variant	c.-25C > T	16.91	not maternal	
**P02**	*BLMH*	17:28575944	G/A	3_prime_UTR_variant	c.*91C > T	15.7	N/A	
**P02**	*CARM1*	19:11030266	C/T	splice_region_variant	c.1021–5C > T	15.69	not maternal	
**P10**	*IKZF3*	17:37949260	C/T	intron_variant	c.164-74G > A	15.38	maternal	
**P10**	*PTPN1*	20:49126913	G/A	5_prime_UTR_variant	c.-152G > A	17.68	N/A	
**P10**	*SEPSECS*	4:25161856	G/A	intron_variant	c.114 + 22C > T	16.15	not maternal	
**P15**	*HES7*	17:8026335	G/A	intron_variant	c.138 + 14C > T	15.59	not maternal	
**P15**	*CNGB3*	8:87755859	C/T	5_prime_UTR_variant	c.-4G > A	15.68	not maternal	
**P18**	*DLGAP1*	18:3874312	T/C	intron_variant	c.957 + 4800A > G	16.45	N/A	
**P19**	*FAM65C*	20:49206219	T/C	splice_region_variant	c.2661+4A > G	17.98	not maternal	
**P19**	*BRAF#*	7:140453973	T/A	intron_variant	c.1741 + 14A > T	17.02	not maternal	
**P12**	*TCF7*	5:133451301	C/T	5_prime_UTR_variant	c.316 + 223C > T	15.81	*de novo*	yes [*TCF7* VUS (c.1060C > G)]
**P25**	*UCP3*	11:73715453	G/T	intron_variant	c.643 + 76C > A	19.81	N/A	
**P25**	*ITGA7*	12:56094605	C/T	intron_variant	c.670 + 78G > A	16.72	N/A	
**P25**	*PPP1R12A*	12:80172901	G/C	intron_variant	c.2956–521C > G	18.05	N/A	
**P25**	*NIN*	14:51297660	G/C	intron_variant	c.-76 + 64C > G	19.02	N/A	
**P25**	*CREBBP#*	16:3811652	G/A	intron_variant	c.3251–2679C > T	16.15	N/A	
**P25**	*CDK12*	17:37618316	G/A	5_prime_UTR_variant	c.-9G > A	18.03	N/A	
**P26**	*PLK3*	1:45269400	C/T	intron_variant	c.1164 + 37C > T	15.47	not maternal	
**P26**	*SERPINI2*	3:167184804	A/T	intron_variant	c.508 + 39T > A	17.66	not maternal	
**P26**	*GFRA2*	8:21645850	G/C	5_prime_UTR_variant	c.-179C > G	18.32	not maternal	
**P26**	*ZNF462*	9:109692049	A/G	intron_variant	c.5847+9A > G	18.73	not maternal	

#known/candidate cancer predisposition genes; *given according to GRCh37; N/A: not available.

**FIGURE 4:**
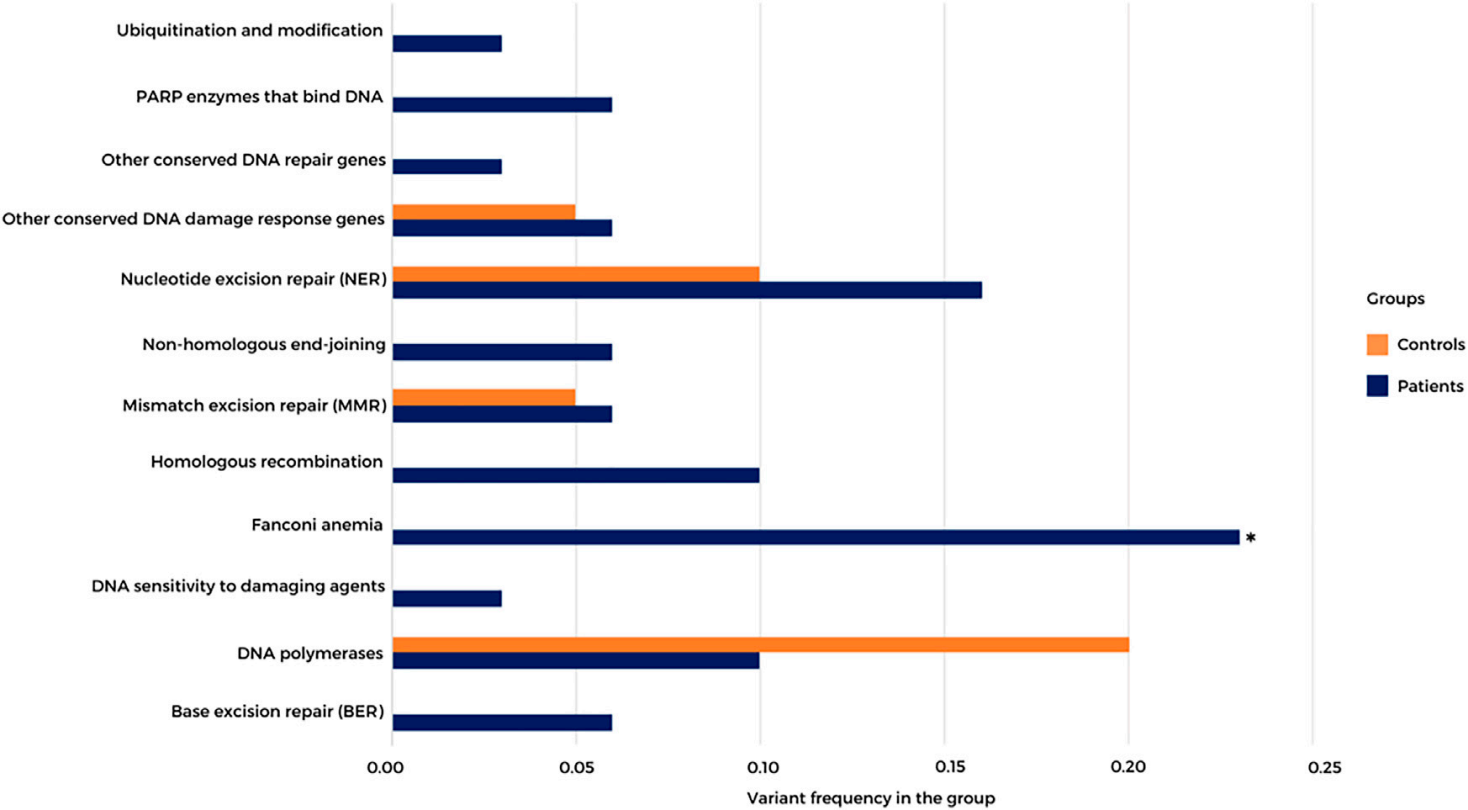
Frequency of high-quality rare germline coding variants mapped to DNA repair genes in HB patients and a control group. A list of 220 DNA repair genes distributed in 16 categories was analyzed; the 12 categories with variants detected in either patients or controls are represented. PARP—poly (ADP-ribose) polymerase. *p value 0.0338; Fisher’s test.

Using the exome data of HB patients and a healthy control group (*n* = 19, data not shown), we inspected a list of 220 DNA repair genes (distributed in 16 categories, including several *bona fide* CPGs; https://www.mdanderson.org/documents/Labs/Wood-Laboratory/human-dna-repair-genes.html), searching for rare LoF and missense variants with high *in silico* damage prediction (5 or more algorithms; (Supplementary Table S8); Figure 4 shows the frequency of rare damaging variants detected in 12 DNA repair categories in both controls and patients. Thirty-four heterozygous variants mapped to DNA repair genes were observed in 21 patients (70%), while nine heterozygous variants were detected in nine healthy controls (47%). Although not statistically significant, there was an apparent excess of damaging variants mapped to DNA repair genes in patients. Moreover, rare damaging variants affecting specific DNA repair gene categories, such as ubiquitin modification, poly (ADP-ribose) polymerase (PARP) enzymes that bind to DNA, nonhomologous end-joining, homologous recombination (*BRCA1, EME2, SPIDR,* and *RAD54L*), Fanconi anemia genes (*BRCA2, FANCA, BRIP1, SLX4, FANCD2,* and *FAAP24*), genes associated with DNA sensitivity to damaging agents, and base excision repair, were observed only in patients. Significant enrichment for rare damaging variants mapped to Fanconi anemia genes was detected in the group of patients (*p* value 0.0338; Fisher’s test).

### Germline Copy Number Variation (CNV) and Regions of Homozygosity

Assessing germline CNVs using exome data, seven rare copy number changes were detected, four with clinical relevance, all of which were validated by CMA (Supplementary Figure S2). The encompassed genomic regions did not include CPGs or genes associated with the patients’ phenotypes (Table 5). A pathogenic Y chromosome aneuploidy was detected in P19 (Jacob syndrome 47, XYY). P21 and P28 paternally inherited 15q11.2 copy number gain and loss, respectively, which are classified as risk factors for neurodevelopmental disorders, mainly global delay, and intellectual disability.

**TABLE 5:** Rare germline CNVs containing coding regions and copy neutral events (>10 Mb) detected by whole-exome sequencing.

ID	Genomic Coordinates (GRCh37)	Cytoband	Lenght (pb)	Event	Classification
P01	chr14:39878443-50253620	14q21.1q21.3	10375178	ROH	N/A
P02	chr12:13243647-26779469	p13.1p11.23	13535823	ROH	N/A
P04	chr3:155563877-169430359	q25.31q26.2	13866483	ROH	N/A
	chr5:82871986-93727017	q14.3q15	10855032	ROH	N/A
P05	chr3:148784725-159539084	q24q25.33	10754360	ROH	N/A
P16	chr1:30815910-44569110	p35.2p34.1	13753201	ROH	N/A
	chr1:100623391-117602935	p21.2p13.1	16979545	ROH	N/A
	chr4:38830037-49064655	p14p11	10234619	ROH	N/A
	chr5:49386907-76121142	q11.1q13.3	26734236	ROH	N/A
	chr7:27135316-43664239	p15.2p13	16528924	ROH	N/A
	chr8:30975883-43822511	p12p11.1	12846629	ROH	N/A
	chr8:46706294-91982327	q11.1q21.3	45276034	ROH	N/A
	chr11:75567951-93408344	q13.5q21	17840394	ROH	N/A
	chr13:77527773-113153328	q22.3q34	35625556	ROH	N/A
	chr16:55222478-65019075	q12.2q21	9796598	ROH	N/A
	chr16:69198915-89289425	q22.1q24.3	20090511	ROH	N/A
	chr17:1-5308028	p13.3p13.2	5308028	ROH	N/A
	chr17:53853305-65025445	q22q24.2	11172141	ROH	N/A
	chr18:1339613-12110120	p11.32p11.21	10770508	ROH	N/A
	chr18:60985801-78077248	q21.33q23	17091448	ROH	N/A
P18	chr1:165649300-197321158	q24.1q31.3	31671859	ROH	N/A
	chr3:75431870-91000000	p12.3q11.1	15568131	ROH	N/A
	chr6:90497518-105697034	q15q21	15199517	ROH	N/A
	chr8:2055270-24208142	p23.3p21.2	22152873	ROH	N/A
	chr13:28538303-42485700	q12.2q14.11	13947398	ROH	N/A
	chr13:52351088-95062039	q14.3q32.1	42710952	ROH	N/A
	chr16:78439762-90354753	q23.1q24.3	11914992	ROH	N/A
P19	chrY:2787139-26671311	Yp11.2q12	23884173	aneuploidy	pathogenic (Jacob syndrome), *de novo*
P18	chr14:64193920-64700120	q23.2	506,201	CN Gain	variant of uncertain significance (VUS), maternal
P07	chr11:93063308-93583708	q21	520,401	CN Gain	likely benign
P09	chr1:19,849,733-20154295	p36.13	304,563	CN Gain	likely benign
P15	chr21:40555024-40569195	q22.2	14172	CN Loss	likely benign
P21	chr15:22612422-23288771	q11.2	676,350	CN Loss	risk factor neurodevelopmental disease (https://search.clinicalgenome.org/kb/gene-dosage/region/ISCA-37448/OMIM #615656 CHROMOSOME 15q11.2 DELETION SYNDROME), paternal
P28	chr15:22740164-23685606	q11.2	945,443	CN Gain	risk factor neurodevelopmental disease (https://search.clinicalgenome.org/kb/gene-dosage/region/ISCA-37448), paternal
P30	chr1:39878521-39879833	p34.3	1,313	CN Gain	likely benign

ROH: copy-neutral region of homozigozity; N/A: not applicable.

Regions of homozygosity (ROH) were also investigated in the germline exome data (Table 5). The individuals P05, P16, and P18 harbor several large ROH, evidencing parental consanguinity. Germline 11p15.5 loss of heterozygosity (LOH) was not observed in any of the patients by exome analysis. However, in one of the patients (P21), a somatic 11p LOH event was detected in the respective tumor sample (Supplementary Figure S3).

## Discussion

We explored the clinical features and spectrum of rare germline variants possibly associated with cancer risk in 30 children who developed HB. We report here an enrichment of birth defects in HB patients (37%), mainly craniofacial (17%, including craniosynostosis) and kidney (7%) anomalies, as well as Hirschsprung disease (7%) and nail dysplasia (7%). There is a growing robust bulk of evidence emphasizing that various birth defects occur in association with a significant increase in the risk of developing childhood cancer (Krivit and Good, 1957; Agha et al., 2005; Johnson et al., 2017). Recent studies also supported an increased risk of pediatric cancer in individuals with birth defects unrelated to chromosomal abnormalities or known genetic syndromes (Norwood et al., 2017; Siegel et al., 2017). HB are reported to occur in association with a wide variety of congenital abnormalities (Narod et al., 1997; Ansell et al., 2005; Scollon et al., 2017), especially craniosynostosis and renal anomalies (de Camargo et al., 2011), which we also observed in this study. Two recent studies used large population-based linkage data to investigate the association between birth defects and childhood cancer, showing that HB are more prevalent in children with birth defects (5.0% in this group compared to 1.3% in children with cancer; Schraw et al., 2020) and frequently occurs in association with craniosynostosis (Lupo et al., 2019). It is interesting that we found in this group of patients a marginally significant result suggesting a tendency of high-risk tumors to develop in patients who also have craniofacial defects. In addition, a large case-control study confirmed the association between HB and kidney and bladder abnormalities (Venkatramani et al., 2014). In this study, we also described the cases of two patients with Hirschsprung disease and HB, a condition with extensive genetic heterogeneity (Sergi et al., 2017; Heuckeroth, 2018); most Hirschsprung disease cases are sporadic (approximately 70%), while a smaller portion of patients have other associated congenital anomalies*.* However, pathogenic, or likely pathogenic variants mapped to morbid OMIM genes that could explain the syndromic phenotypes of our patients were not detected, reinforcing that, despite the long-standing knowledge about the intersection between biological pathways of cancer and development, the etiology of most of these associations in specific cases remains unknown.

There is a recognized sexual dimorphism in HB, with increased prevalence in males (Spector and Birch, 2012; Williams and Spector, 2019), and this study also reflected this tendency. We investigated whether this sex bias could be explained by an increased burden of rare damaging variants in one of the sexes; however, significant differences were not detected, suggesting that most likely environmental factors modulate the sexual dimorphism in HB prevalence.

In addition to SNV/indel variants, germline CNVs were already reported as causally related to cancer (Krepischi et al., 2012), but pathogenic CNVs affecting known/candidate CPGs were not detected in this study. One patient (P19) carried a gain of an entire Y chromosome, which is associated with an increased risk of learning disabilities, behavioral disturbances, and other clinical signs (Lalatta et al., 2012; Bardsley et al., 2013). Moreover, two 15q11.2 CNVs, considered risk factors for neurodevelopmental disorders (Butler, 2017; Rafi and Butler, 2020), were identified in two HB patients (P21 and P28). Only one patient (P28) presented an unspecific clinical feature related to the condition associated with its CNV (developmental delay). However, all these detected copy number alterations are known to present incomplete penetrance and variable expressivity. Importantly, germline 11p15.5 LOH, a common molecular mechanism resulting in epigenetic alterations known to increase the HB risk (Carrillo-Reixach et al., 2020; Fiala et al., 2020) as not detected in any of the patients.

Germline mutations have been detected in 8–18% of children and adolescents with cancer (Zhang et al., 2015; Akhavanfard et al., 2020; Capasso et al., 2020; Newman et al., 2021), and the most prevalent CPGs reported to be mutated are *TP53, APC, BRCA2, NF1, PMS2, RB1,* and *RUNX1* (Zhang et al., 2015). In a very recent work about the spectrum of germline mutations in childhood cancer, most of the mutations (55%) were mapped to genes not previously associated with the patient’s tumor type (Newman et al., 2021); this work included a small number of HB patients (3 patients) with no pathogenic or likely pathogenic variants. Our findings revealed a high burden of damaging germline variants mapped to CPGs, with only 40% of these patients presenting a family history of cancer. Most of the variants affected autosomal-dominant CPGs not previously linked to HB, since only one mutation was detected in a known gene of HB development (*APC*), in a familial context of relatives diagnosed with FAP/colon cancer which segregated with the *APC* mutation. Interestingly, our data showed a clear predominance of CPGs related to gastrointestinal/colorectal cancer risk, such as familial adenomatous polyposis one and 2 (*APC* and *MUTYH*), nonpolyposis colorectal cancer hereditary types 1 (*MSH2*) and 6 (*TGFBR2*), Li-Fraumeni syndrome 2 (*CHEK2*), and Diamond-Blackfan anemia 1 (*RPS19*). Furthermore, the CPGs *DROSHA* and *VHL*, with putative P/LP variants, are known to be associated with renal cancer. Finally, a LoF variant was identified in the DNA repair gene *ERCC5*, in which homozygous mutations increase the skin cancer risk, and a damaging missense variant was mapped to *FAH* and causes an autosomal-recessive disorder characterized by progressive liver disease with increased liver cancer risk. Compared to previous pan-cancer studies, although not in HB patients, the findings of germline variants in *APC, CHEK2, ERCC5, MSH2, MUTYH,* and *VHL* were also detected in our work (Zhang et al., 2015; Ma et al., 2018a; Grobner et al., 2018; Newman et al., 2021).

We also observed numerous rare damaging VUS in known or candidate CPGs. Variants mapped to the *ATM, BRCA2, COL7A1, DHCR7, DOCK8, FANCD2, FANCM,* and *GLI3* genes were observed in more than one HB patient. In particular, the same *DHCR7* VUS (c.988G > A) was spotted in two patients (P09, born with only one functional kidney, and P26), both diagnosed with low-risk epithelial fetal HB; this gene encodes an enzyme that catalyzes the conversion of 7-dehydrocholesterol to cholesterol (Fitzky et al., 1998) and is a candidate for familial breast cancer in *BRCA1-*and *BRCA2*-negative breast cancer families (Shahi et al., 2019), in a recessive mode of action. As in our study, VUS mapped to *MET, ATM, SLX4*, and *FANCA* were also reported in HB patients in a recently published large study (Newman et al., 2021).

Strong support exists for the hypothesis that monoallelic CPG mutations confer an increased risk of cancer for adult carriers, while biallelic carriers would have a high risk of childhood cancer (Rahman and Scott, 2007). More recently, large studies have shown that variants in heterozygosity affecting recessive genes can also increase the predisposition to pediatric cancer (Savary et al., 2020), and a second hit in the tumor, such as a loss of heterozygosity or an inactivation of the second allele, has been observed in some of these patients. A highlight has been given to the role of germline monoallelic variants in cancer predisposition in genes involved in the recognition and repair of DNA damage, such as the *ATM*, *PALB2*, and Fanconi anemia genes (Thompson et al., 2005; Renwick et al., 2006; Antoniou et al., 2014; Helgason et al., 2015; Rebbeck et al., 2015; Esteban-Jurado et al., 2016; Esai Selvan et al., 2019). Since 1971 (Swift et al., 1971), it was proposed that individuals who were heterozygous for the Fanconi anemia genes might be at increased risk of cancer, and the premise would be that a modest reduction in the DNA repair efficiency could lead to tumor development. In our work, we detected relevant monoallelic variants in CPGs associated with recessive clinical conditions (*FAH, ERCC5,* and *MUTYH*) and in DNA repair genes (*MSH2, CHEK2, ERCC5,* and *MUTYH*). Recently, it has been shown that carriers of monoallelic loss of function *MUTYH* germline variants are at a higher risk of developing cancer, especially in tumors with frequent loss of heterozygosity events, such as adrenal adenocarcinoma, although the overall risk is still low (Barreiro et al., 2022); unfortunately, tumor sample of Patient P28, who carry a germline heterozygous *MUTYH* variant, was not available to check if loss of heterozygosity was present as a second hit, as suggested by authors. In addition, we observed a general enrichment of heterozygous germline damaging VUS affecting DNA repair genes related to Fanconi anemia, nucleotide and base excision repair, and homologous recombination repair. Sequencing the tumors of the carriers could contribute to changing the classification of such variants (Newman et al., 2021) and clarifying the role of these variants in cancer predisposition.

One of the most interesting findings of this study was the disclosure of rare germline damaging variants affecting genes linked to liver differentiation and function, such as *FAH*, that cause a recessive disorder related to hepatocellular carcinoma risk. Several rare damaging variants were observed in genes of the cytochrome P450 (CYP) family, including one pathogenic heterozygous variant in *CYP21A2* (OMIM #201910 ADRENAL HYPERPLASIA, CONGENITAL, DUE TO 21-HYDROXYLASE DEFICIENCY, a recessive condition associated with testicular neoplasia in adults—P22) and two heterozygous pathogenic *CYP1B1* variants (OMIM #617315, #231300; recessive conditions) detected in different patients (P03 and P30). Ten rare variants mapped to *CYP1A1* (one LoF and nine missense variants), which is associated with primary liver metabolism, were present in eight patients (26%). *CYP1A1* encodes a xenobiotic-metabolizing enzyme acting in the placenta (Yan et al., 2005; Suter et al., 2010; Stejskalova and Pavek, 2011), as well as in several drugs and compounds widely used in pharmacotherapy (Willis et al., 2018; Kapelyukh et al., 2019) or present in the diet (Ito et al., 2007). *CYP1A1* expression is transcriptionally regulated through the AhR receptor (Kawashiro et al., 1998; Delescluse et al., 2000; Kamenickova et al., 2013) and various exogenous AhR binders, such as nitrosamines, polycyclic aromatic hydrocarbons, polychlorinated biphenyls, and halogenated dioxins, can be found in products from combustion processes, such as chimney soot, grilled food, and cigarette smoke, or as the product of incineration waste (Larigot et al., 2018; Lu et al., 2020; Schanz et al., 2020; Shahin et al., 2020). Elevated *CYP1A1* activity through AhR activation in the placentas of female smokers has been associated with pregnancy complications, including low birth weight (Stejskalova et al., 2011), a known risk factor for HB. In our previous study (Aguiar et al., 2020), in which we investigated the mutational burden of HB, a mutational signature like the COSMIC 29 signature was detected, which was initially observed only in oral gengivo-buccal squamous cell carcinoma, which develops in individuals with the habit of chewing tobacco. Currently, this mutational signature has also been detected in patients with lung, thyroid, bladder, biliary, breast, pancreas, liver, and kidney cancer and can be linked to nitrosamine exposure (Alexandrov et al., 2015). It is interesting to notice that two of the patients carrying germline *CYP1A1* variants developed HBs with predominance of S29. Therefore, we can speculate that a defective response during pregnancy to xenobiotic exposure, such as nitrosamines, could be linked to *CYP1A1-*damaging germline variants, increasing the risk for HB development, and resulting in the mutational signature we previously found in HB.

In conclusion, our major findings in HB patients were the detection of germline variants in CPGs associated with gastrointestinal/colorectal and renal cancer risk, not always linked to a familial history of cancer; the enrichment of monoallelic variants in CPGs and DNA repair genes; and a high frequency of birth defects. One limitation of our study is the small size of the group of patients, which precludes robust statistical analysis and demands further evaluation of germline data of larger cohorts; however, HB is indeed an ultrarare condition, and this is the largest assessment of germline variants in HB patients to date. Another point that deserves to be mentioned is that we did not investigate germline 11p15.5 epimutations, which are known to be associated with increased HB risk; this limitation was only partially overcome by the investigation of 11p15 regions of homozygosity in exome data. Finally, most studies of HB tumors are from North America, Europe, and Asia, and this work is pioneering in South America and contributes to elucidating the genetic architecture of HB risk. Further validation of the genes highlighted as HB germline risk factors in other cohorts can provide new insights regarding HB development.

## Data Availability

The original contributions presented in the study are included in the article/Supplementary Material, further inquiries can be directed to the corresponding authors. Data is submitted to the https://www.ncbi.nlm.nih.gov/clinvar/ with identifier: SUB11168694 (SCV002103098 - SCV002103150).

## Abbreviations

CNV, Copy number variant; CPGs, Cancer predisposing genes; FA, Fanconi anemia; FAP, Familial adenomatous polyposis; FFPE, Formalin-fixed paraffin-embedded; HB, Hepatoblastoma; IGV, Integrated Genomics Viewer; LoF, Loss of function; LP, Likely pathogenic; P, Pathogenic; VUS, Variant of uncertain significance; WES, Whole-exome sequencing.
