# Supplementary material for: Unraveling the Genetic Architecture of Hepatoblastoma Risk: Birth Defects and Increased Burden of Germline Damaging Variants in Gastrointestinal/Renal Cancer Predisposition and DNA Repair Genes
Source: Front Genet. 2022 Apr 12;13:858396. doi: 10.3389/fgene.2022.858396 (PMC9039399; doi:10.3389/fgene.2022.858396)
Supplement: Supplementary file 1 [file DataSheet2.pdf]

# SUPPLEMENTARY FIGURES

**Unraveling the genetic architecture of hepatoblastoma risk: birth defects and increased burden of germline damaging variants in gastrointestinal/renal cancer predisposition and DNA repair genes**

Talita Aguiar[1,2,3*], Anne Teixeira[1,2*],Marília O. Scliar[2], Juliana Sobral de Barros[1,2], Renan B. Lemes[1,2], Silvia Souza[1,2], Giovanna Tolezano[1,2], Fernanda Santos[4], Israel Tojal[5], Monica Cypriano[6], Silvia Regina Caminada de Toledo[6], Eugênia Valadares[7], Raquel Borges Pinto[8], Osvaldo Afonso Pinto Artigalas[9], Joaquim Caetano de Aguirre Neto[10], Estela Novak[11,12], Lilian Maria Cristofani[11], Sofia M Miura Sugayama[13], Vicente Odone[11], Isabela Werneck Cunha[14], Cecilia Maria Lima da Costa[4] , Carla Rosenberg[1,2], Ana Krepischi[1,2#]

1. Department of Genetics and Evolutionary Biology, Institute of Biosciences, University of São Paulo, São Paulo, Brazil.
2. Human Genome and Stem Cell Research Center, Institute of Biosciences, University of São Paulo, São Paulo, Brazil.
3. Columbia University Irving Medical Center, New York, NY, USA
4. Department of Pediatric Oncology, A. C. Camargo Cancer Center, São Paulo, Brazil.
5. International Center for Research, A. C. Camargo Cancer Center, São Paulo, Brazil.
6. GRAACC- Grupo de Apoio ao Adolescente e Criança com Câncer, Federal University of São Paulo, São Paulo, Brazil
7. Benjamim Guimarães Foundation - Department of Pediatrics Hospital da Baleia, Belo Horizonte, Minas Gerais, Brazil.
8. Department of Genetics, Hospital da Criança Conceição, Hospitalar Conceição Group, Porto Alegre, Rio Grande do Sul, Brazil.
9. Department Pediatric Gastroenterology, Hospital da Criança Conceição, Hospitalar Conceição Group, Porto Alegre, Rio Grande do Sul, Brazil.
10. Paediatric Haemato-oncology, Hospital Santa Casa de Belo Horizonte, Belo Horizonte, Brazil.

11. Pediatric Cancer Institute (ITACI) at the Pediatric Department, São Paulo University Medical School, São Paulo, Brazil.

12. Molecular Genetics – Foundation Pro Sangue Blood Center of São Paulo.

13. Department of Pediatric, Faculty of Medicine of the University of São Paulo, São Paulo, Brazil.

14. Department of Pathology, Rede D'OR-São Luiz, São Paulo, Brazil.

***These authors contributed equally to this work.**

**#Corresponding author:**

Ana Cristina Victorino Krepischi

Department of Genetics and Evolutionary Biology - Institute of Biosciences,

The University of São Paulo, São Paulo, Brazil

Phone: 55 11 3091 7573

e-mail: ana.krepischi@ib.usp.br

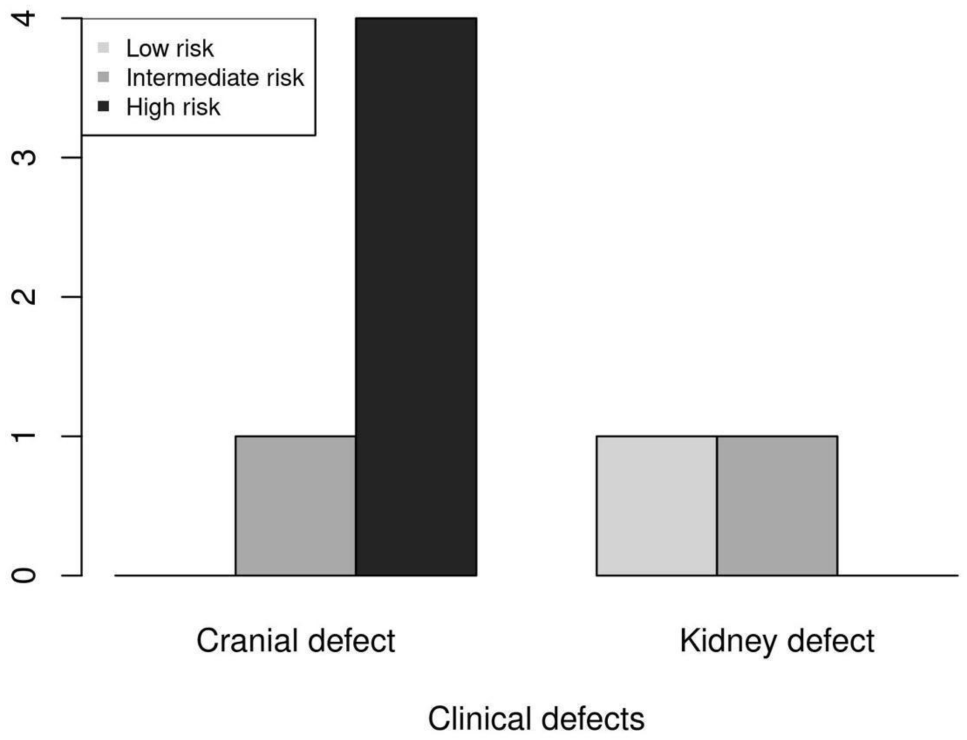


**Supplementary Figure 1. Distribution of clinical conditions according to hepatoblastoma risk stratification.** The y-axis represents the number of patients in each group (low, intermediate, and high risk). There is a tendency of patients with risk of HB present cranial defects than the ones with kidney defects (marginally significant p-value of 0.10 from fisher exact test).

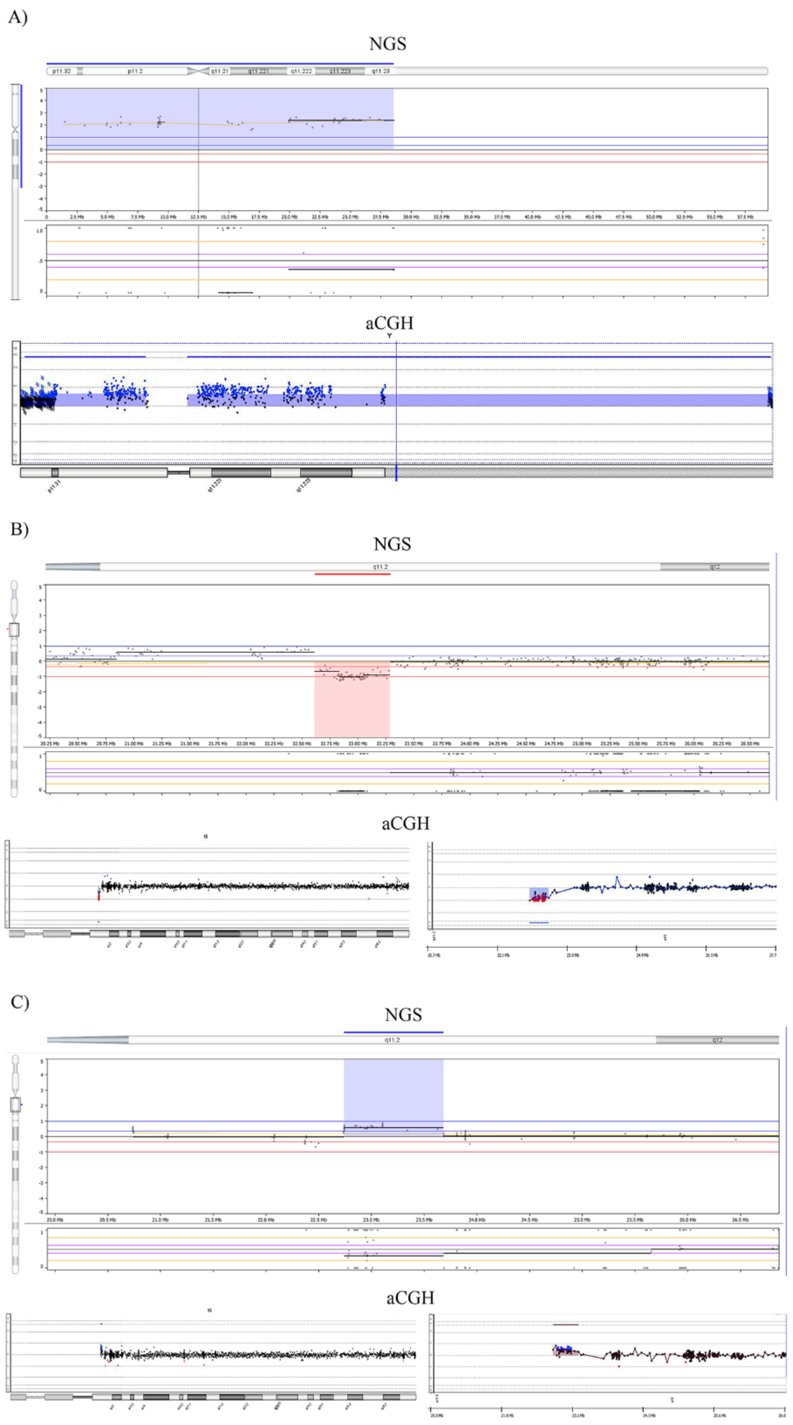


**Supplementary Figure 2. CNV profiles of the three clinically relevant copy number changes observed in Patients P19, P21 e P28.** Detected CNVs are presented in each case showing the exome data (NGS) above and the CMA validation (aCGH) below. Images were extracted from the software Nexus Copy Number 9 (Biodiscovery).

**A)** a pathogenic aneuploidy (the gain of an entire chromosome Y, in blue) identified in P19, resulting in a XXY molecular karyotype.

**B)** and **C)** shows the CNVs at the 15q11.2 recurrent region detected in P21 (deletion, in red) and P28 (duplication, in blue), respectively; in the CMA data, at left is depicted the entire chromosome 15 and, at right, the15q11.2 region in detail.

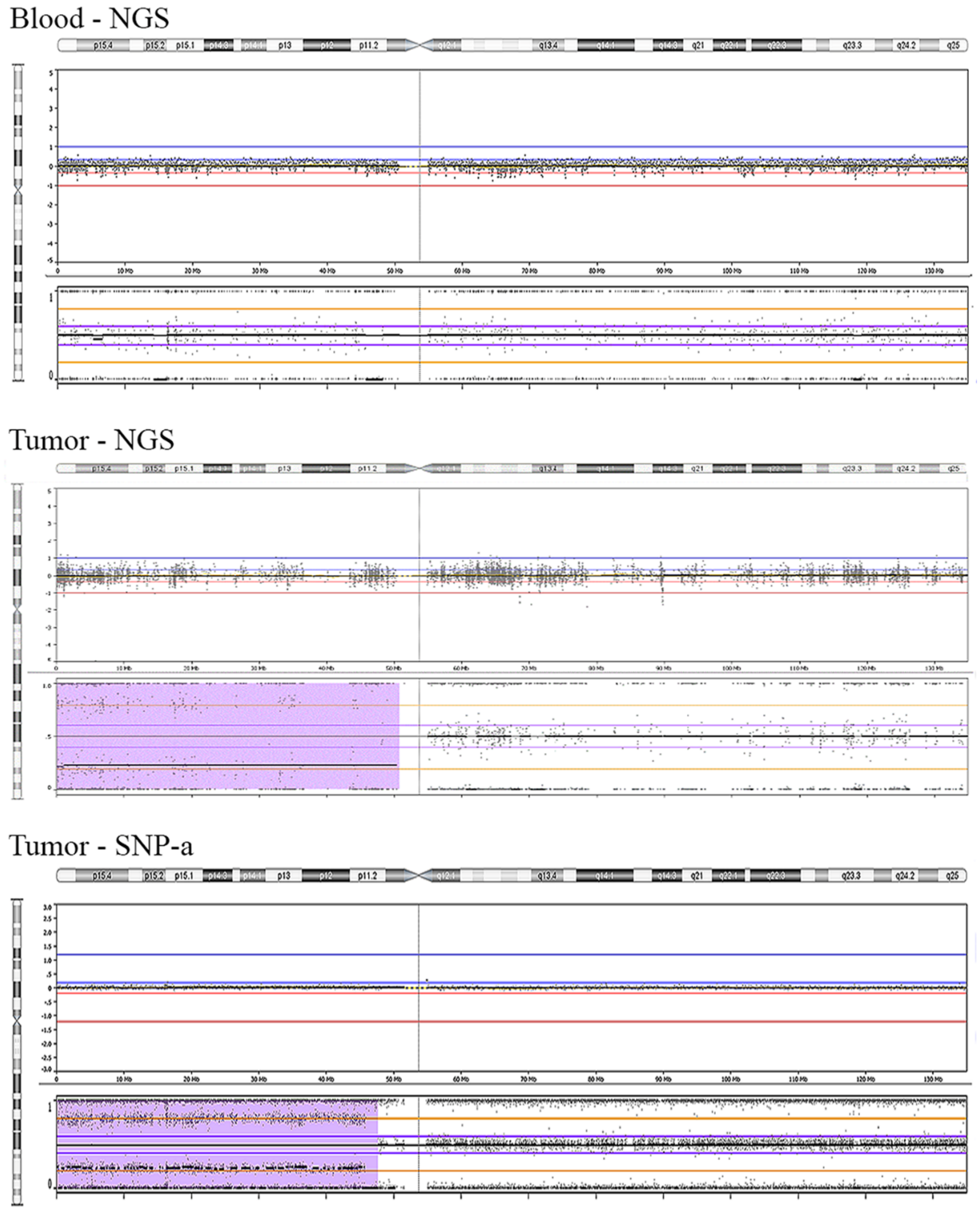


**Supplementary Figure 3. Somatic acquisition of loss of heterozygosity (LOH) at 11p in P21's tumor.**

In the three images, CNV data is plotted in the upper panel, and the lower panel presents the BAF plot. The first image shows the chromosome 11 profile derived from exome data (NGS) of a genomic sample extracted from the blood of Patient P21; it is possible to verify the presence of heterozygosity in the BAF plot. The two images below are identified

as Tumor and they present exome (NGS) and SNP-array (SNP-a) data obtained from a tumoral sample of the Patient 21, evidencing the occurrence of a somatic event of 11p LOH in the BAF plot (purple colored segment). Images were extracted from the software Nexus Copy Number 9 (Biodiscovery).
